# Chronic Spontaneous Urticaria and Type 1 Diabetes Mellitus—Does Quality of Life Impairment Always Reflect Health Danger?

**DOI:** 10.3390/jcm9082505

**Published:** 2020-08-04

**Authors:** Zenon Brzoza, Katarzyna Nabrdalik, Lukasz Moos, Hanna Kwiendacz, Karina Badura-Brzoza, Hanna Jarolim, Katarzyna Kapeluszna, Janusz Gumprecht

**Affiliations:** 1 Department of Internal Medicine with Division of Allergology, Institute of Medical Sciences, University of Opole, 45-401 Opole, Poland; lukasz.moos@uni.opole.pl (L.M.); katarzyna.kapeluszna@uni.opole.pl (K.K.); 2 Department of Internal Medicine, Diabetology and Nephrology, Faculty of Medical Sciences in Zabrze, Medical University of Silesiain Katowice, 40-055 Katowice, Poland; nabrdalik.katarzyna@gmail.com (K.N.); hanna.kwiendacz@gmail.com (H.K.); hjarolim@onet.pl (H.J.); jgumprecht@sum.edu.pl (J.G.); 3 Department of Psychiatry in Tarnowskie Góry, School of Medicine with the Division of Dentistry in Zabrze, Medical University of Silesia, 40-055 Katowice, Poland; kbbrzoza@sum.edu.pl

**Keywords:** chronic urticaria, diabetes, quality of life

## Abstract

Background and aims: Chronic spontaneous urticaria (CSU) and diabetes mellitus type 1 (T1DM) may compromise the quality of life (QoL). We decided to compare the QoL of T1DM patients to those suffering from CSU. Materials and methods: Sixty-six patients with well-controlled T1DM (male 52%) in the mean age of (SD) 36.3 (11.09) years and 51 patients with CSU (male 33%) in the mean age of (SD) 35.8 (8.53) years were enrolled in this observational study. All the participants completed a Short-Form 36 (SF-36) QoL. Results: The QoL related to social functioning was significantly worse among CSU patients. There were differences related to gender found in the group of patients with T1DM—where men tended to declare a better quality of life than women (*p* = 0.015)—especially in the area of energy/fatigue and pain. It appeared that due to physical and emotional problems occurring in married patients, the QoL is lower in T1DM group in comparison to the CSU one. Conclusions: The patients with CSU presented significantly worse social functioning compared to the ones with T1DM. This fact proves the QoL impairment level is not always related to the level of health danger. The differences in the QoL related to gender and marital status found among T1DM patients point to the necessity for further exploration in a larger group of patients. Due to the fact that optimal disease management should ensure patient’s good emotional well-being, there is a need for additional psychological and social care for patients from those two groups.

## 1. Introduction

Chronic spontaneous urticaria (CSU) is diagnosed based on the clinical symptoms observed over a period of time (spontaneous occurrence of itchy wheals and/or angioedema for at least 6 weeks) [1]. In the general population, the prevalence of CSU is estimated to be around 0.5–5% and it is more frequently seen in females (female-to-male ratio about 2:1) [2,3,4]. According to the World Health Organization (WHO), the rate of diabetes mellitus type 1 (T1DM) is increasing by 3% per year in high-income countries [5]. In relation to young people < 20 years of age, according to the 8th Edition of the International Diabetes Federation (IDF) Diabetes Atlas, it is estimated that the number of patients with T1DM in this age category is 1,106,500 million worldwide [6]. The prevalence of T1DM is highest in Scandinavia and lowest in Asia and Latin America. T1DM is more common in males than in females. In populations of European origin, the male-to-female ratio is greater than 1.5:1 [5,7]. Both T1DM and CSU are lifelong diseases which may compromise the quality of life (QoL) and the majority of patients are young and socially active [2,5,8]. T1DM is the result of the interaction between genetic predisposition and environmental factors. When properly treated, T1DM in many patients is asymptomatic; however, the management of the disease and treatment methods are the main factors that might impact the QoL. There are many rules to follow for T1DM patients, such as: lifelong insulin therapy—most patients usually require at least 4 injections of insulin daily and frequent self-monitoring of blood glucose levels-up to 6–10 times daily and diet control-including education about how to adjust the timing, size, frequency, and composition of meals [5,9].

Although CSU does not require so much attention from the patient in terms of self-control and troublesome treatment methods—it is associated with chronic symptoms such as spontaneous recurrent bouts of wheals and pruritus that compromise the QoL in different areas of everyday activities [2,10]. The treatment of CSU is focused mainly on achieving an acceptable level of remission. Unfortunately, achieving satisfying treatment results is not being seen as often as expected. There is an increasing number of patients resistant to the standard treatment regimens of CSU [11]. They require extended diagnostics, followed by a long-term treatment process, which, at the end of treatment, may be unsuccessful. CSU influences the emotional and the psychological aspects of life and has the potential to impair one’s functioning in society, including social contacts and work activity [1]. The QoL impairment in CSU appears to be comparable to that of coronary artery disease and severe asthma, but it is lower than in other skin conditions, namely psoriasis, acne, contact dermatitis, or vitiligo [12,13,14,15,16,17]. 

The QoL measurement of CSU is crucial and recommended by current guidelines in order to make the best treatment decisions and to document the changes in the QoL during the process of the therapy [1]. 

There were also many analyses performed that were related to the QoL of patients with T1DM, mainly in relation to other aspects of the disease, such as complications, glycemic control or diabetes duration [18,19,20,21,22,23,24,25]. However, the QoL among patients with T1DM, who, if properly managed, have no visible signs of the disease, has never been compared to a disease like CSU, where the QoL can be obviously reduced for at least several reasons related to visible skin problems e.g., social isolation [26,27].

As mentioned above, there are various disease-specific QoL measures both for urticaria and for T1DM, but to compare CSU and T1DM a generic one is needed [2,28,29]. Short-Form 36 (SF-36) has been proven useful in comparing general and specific populations of different diseases, including skin disorders [30]. According to Speight et al. [28], who has reviewed instruments used to measure the QoL in diabetes, Short-Form 36 (SF-36) “is reasonable choice if a generic health status measure is needed”.

Patients with T1DM have no visible signs of the disease and the disease itself causes no bodily pain or discomfort. However, in order to obtain good glycemic control, it requires patient’s involvement in treatment processes with multiple daily blood glucose measurements and insulin injections. It may compromise the QoL to the level observed in chronic skin disease, which causes bodily discomfort. Taking the above information into account, we decided to compare the QoL of T1DM adult patients with good metabolic control of the disease to those suffering from CSU, with the use of SF-36. To our best knowledge, there were no studies performed before that related directly to this issue.

## 2. Material and Methods 

This was a multicenter, questionnaire-based, observational study where we assessed the QoL of patients with T1DM and CSU who routinely visited an outpatient diabetology or allergology clinic, respectively. Inclusion criteria were as follows: aged 18–65 years, T1DM or CSU, and no other concomitant chronic diseases. In relation to T1DM, patients had to have good diabetes control with glycated hemoglobin (HbA1c) in the range of 6.5–7.5%, with rare symptomatic hypoglycemia occurrence, namely less than 2 times a week. Moreover, they had to be treated with functional insulin therapy with the use of pen injectors and declare to perform at least 6 measurements of blood glucose a day. Inclusion criteria for patients with CSU were recurrent symptom manifestation with necessity of constant antihistamine usage. Disease symptom severity was assessed with the Urticaria Activity Score (UAS). The tool analyses the number of wheals and pruritus intensity. We performed a seven-day assessment (UAS7) [31].

To compare the QoL between CSU and T1DM, a SF-36 questionnaire was used. This questionnaire covers 8 domains: mental health (MH), vitality (VT), general health (GH), bodily pain (BP), physical functioning (PF), role limitations due to emotional problems (RE), role limitations due to physical problems (RP) and social functioning (SF). Each domain is scored 0–100—the lower the score, the higher the disability [30]. Additionally, the following characteristics of the patients were studied: age, sex, marital status, education (elementary/secondary/vocational/higher), and duration of the disease.

The statistical analysis was performed using Statistica v 13.1 (Statsoft INC., USA) and analyses included Kruskall-Wallis and U Mann-Whitney tests. A *p* level below 0.05 was considered significant. 

The study was performed in accordance with the Declaration of Helsinki and was approved by the local Bioethics committee (ethical approval number KNW/0022/KB1/18/15). All participants gave written, informed consent to participate in the study. The manuscript was prepared according to Strengthening the Reporting of Observational studies in Epidemiology (STROBE) guidelines [32].

## 3. Results

**Figure 1:**
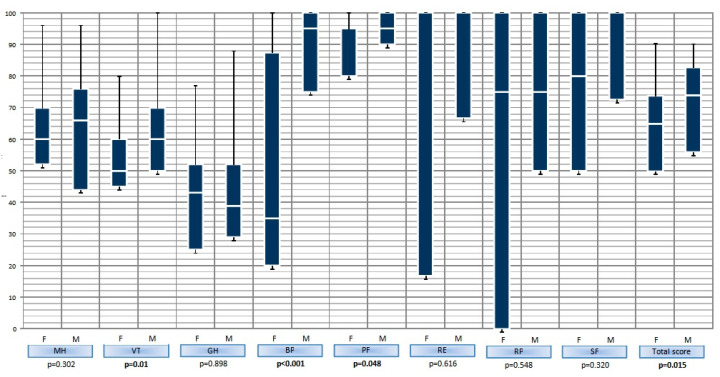
Differences in the quality of life (QoL) in type 1 diabetes mellitus (T1DM) in relation to SF-36 domains. F—female, M—man, mental health (MH), vitality (VT), general health (GH), bodily pain (BP), physical functioning (PF), role limitations due to emotional problems (RE), role limitations due to physical problems (RP), social functioning (SF).

One-hundred and twenty four eligible patients with CSU or T1DM visiting outpatient allergology or diabetology clinics, respectively, were invited to participate in the study, of whom 117 agreed to be enrolled. The study population comprised of adult patients aged 18–59 years diagnosed with CSU (*n* = 51, male 33%) in the mean standard deviation (SD) age of 35.8 (8.53) years and with T1DM (*n* = 66, male 52%) in the mean (SD) age of 36.3 (11.1) years (Table 1). The mean (SD) value of UAS7 in the CSU group was 17.4 (8.17). All of the enrolled patients filled in the SF-36 questionnaire. There was a significant difference in the disease duration between the studied groups (Table 1). A statistically significant difference in the social functioning domain between T1DM and CSU was noticed. CSU patients had a lower QoL in this area (Table 2). Comparing genders revealed that the difference in the mentioned domain was significant in men, but not in women. Women appeared to have impaired QoL within the T1DM group. The differences were seen in the general SF-36 result and also in specific domains like vitality, bodily pain and physical functioning (Figure 1). No gender-related differences were presented in the CSU group.

**Figure 2:**
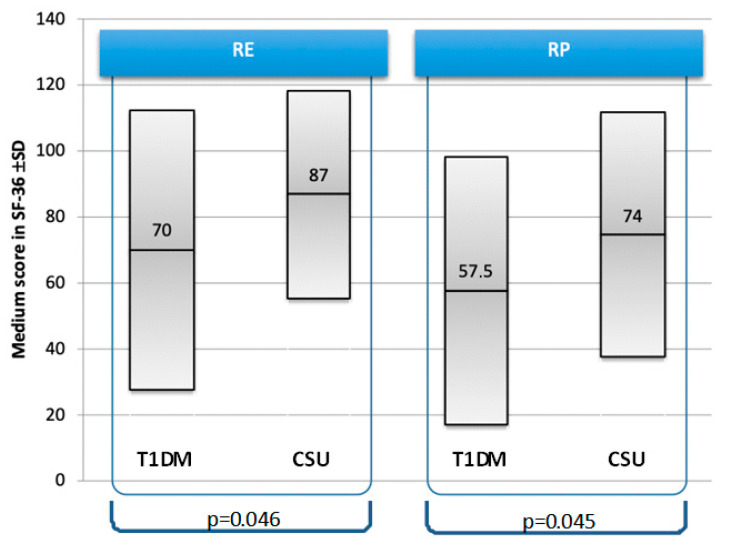
Impairment in the quality of life (QoL) of married patients with type 1 diabetes mellitus (T1DM) and chronic spontaneous urticaria (CSU), referring to SF-36 “role limitations due to emotional problems” (RE) and “role limitations due to physical problems” (RP).

No significant differences were revealed between the groups in regard to education level. On the other hand, in married patients there was a significant difference in the QoL in reference to role limitations due to emotional or physical problems. It appeared that in these domains married patients’ QoL is lower in the T1DM group in comparison to the CSU one (Figure 2).

## 4. Discussion

CSU compromises patients’ QoL in different aspects of everyday life, mainly due to its debilitating symptoms that can last for years [1,2,3,12,13,14,33,34]. Our findings suggest that adult patients with CSU have a worse QoL in social functioning compared to patients with T1DM. This demonstrates that skin disease, although it may not shorten lifespan and does not bring serious health complications as T1DM does (in some cases it might be even life-threatening), nevertheless causes significant impairment to the QoL [35]. It seems to be important to emphasize that the QoL was lower even though the disease duration time was much shorter for urticaria. CSU in this context appears to compromise the QoL from the very beginning. At the same time, a well-educated T1DM patient, knowing how to use insulin, can lead a normal life [36]. The results we present are consistent with the ones performed on children aged 5–16 years with skin disease and other chronic diseases [37]. Beattie et al. [37] found that only diseases such as cerebral palsy, generalized atopic dermatitis, renal disease and cystic fibrosis had a stronger negative impact on the QoL than urticaria, which was followed in descending order by asthma, psoriasis, epilepsy, enuresis, diabetes, localized eczema, alopecia and acne. We have not found any similar publications referring to adult patients or directly comparing CSU and T1DM.

O’Donnell et al. [13] in 1996 investigated the QoL of patients suffering from CSU and from combined CSU and delayed pressure urticaria (DPU). They compared their results to the study of 100 males with coronary heart disease (CHD) awaiting coronary artery bypass grafting, which was performed by Caine et al. [38] in 1991. As a result of this comparison, it was revealed that sleep disruption was a greater problem for patients with urticaria and their QoL was similarly impaired in the terms of energy, social isolation and emotional reaction. Outcomes of our work are in accordance with those mentioned above: social functioning in men with CSU appeared to be worse than in the more dangerous disease, T1DM. As Caine et al.’s [38] study was referring to men only, there is no data comparing the QoL between females suffering from CHD and CSU. We did not find any data directly comparing patients with CHD to CSU patients at the same time (as progress in cardiology may have impact on the QoL of patients with CHD).

It is worth analyzing the data that show differences in the QoL between the gender of patients with T1DM. As presented before, women’s QoL is impaired in the “vitality” (related to energy and fatigue), “bodily pain” and “physical functioning” domains. These findings are similar to other studies examining the QoL of patients with T1DM which all show a poorer QoL in females, both in childhood and in adult life [8,39,40]. Nilsen et al. [40] suggest that this might be due to the fact that complications and distress are more prevalent among women with T1DM. Men with diabetes experience a higher treatment satisfaction and a lower diabetes burden compared to the opposite sex [41]. Moreover, females, regardless of age, are generally more worried, sensitive and have lower self-rated health compared to male counterparts [42,43].

Interestingly, in our data, we found significant differences in the QoL of married patients. Married patients with T1DM have more role limitations due to emotional problems and physical health compared to CSU ones. We suspect it might be connected to the significant difference in the duration of the disease from the time of diagnosis between the groups (mean time for T1DM of nearly 17 years and for CSU of nearly 4 years). According to studies by Costa L et al. [39] and Anderson BJ et al. [8], the QoL of patients with T1DM is worsening with the time since diagnosis and with the increase in glycated hemoglobin (HbA1c), which proves insufficient glycemic control [8,39]. The problems for patients suffering from T1DM change over time, at first, they are bound to treatment regimens and the necessity of self-control, and after years of the disease when complications may occur, in addition to the everyday treatment, they may also have to deal with disease complications [9,39,44,45]. It may also be supposed that the impairment of the QoL seen in married T1DM patients might be related to sexual dysfunction, which tends to be of a greater extent in patients with a long-lasting disease [46,47].

## 5. Conclusions

T1DM causes a constant need for blood glucose control and insulin injections, and CSU causes discomfort and pruritus. They have a completely different clinical picture, yet both of them similarly compromise patients’ general QoL. However, patients with CSU presented significantly worse social functioning in comparison to the ones with T1DM. Impairment in the QoL of patients with CSU is marked from the very beginning of the disease.

Additionally, it is interesting that there were gender differences found in the QoL among patients with T1DM, and this needs further investigation on a larger group of patients. The impairment in the QoL of married T1DM patients also appears to be an interesting area to explore.

Due to the fact that good disease management should ensure patient’s good emotional well-being, there is a need for additional psychological and social care for patients from these two groups. For patients suffering from T1DM, there might be slightly more time for this intervention, as the QoL seems to be lowering along with the disease duration. Nevertheless, for patients with CSU, the support concerning the QoL should be considered from the day the diagnosis is established.

## Figures and Tables

**Table 1:** Basic characteristic of study groups.

	M (%)	F (%)	Age			Duration of the Disease		
			Mean	SD	Range	Mean (Years)	SD	Range
**T1DM (*n* = 66)**	34 (52%)	32 (48%)	36.3	11.1	18–59	16.9	9.45	0.1–42
**CSU (*n* = 51)**	17 (33%)	34 (67%)	35.8	8.53	19–49	3.84	6.84	0.17–30
***p***	0.05		0.80			0.00		

F—female, M—men, T1DM—type 1 diabetes mellitus, CSU—chronic spontaneous urticaria, *p*—significance level, SD—standard deviation.

**Table 2:** Differences in quality of life (QoL) between type 1 diabetes mellitus (T1DM) and chronic spontaneous urticaria (CSU) referring to Short-Form 36 (SF-36) domains: mental health (MH), vitality (VT), general health (GH), bodily pain (BP), physical functioning (PF), role limitations due to emotional problems (RE), role limitations due to physical problems (RP), social functioning (SF), total score (Ts), M—male, F—female.

	T1DM		CSU		*p*
	Median	Range	Median	Range	
MH	62	52–76	64	56–76	0.31
VT	60	45–70	60	45–70	0.64
GH	42.5	28–52	40	30–54	0.55
BP	75	20–100	75	30–100	0.90
PF	95	89–100	95	85–100	0.71
RE	100	66.7–100	100	100–100	0.17
RP	75	0–100	100	50–100	0.14
SF	100	50–100	72.5	37.5–100	0.03
SF-M	100	72.5–100	75	37.5–87.5	0.03
SF-F	80	50–100	72.5	37.5–100	0.31
Ts	69.3	52.1–77.6	73.4	53.7–82.4	0.32
